# Correlation Between the High‐Sensitivity C‐Reactive Protein‐to‐Albumin Ratio and All‐Cause Mortality in Patients With Acute Exacerbation of Chronic Heart Failure

**DOI:** 10.1002/clc.70456

**Published:** 2026-08-24

**Authors:** Xi Gu, Jinli Feng, Pinye Chen, Hao Wang, Tao Rui, Junfang Guo

**Affiliations:** ^1^ Department of Cardiology Jiangsu University Affiliated People's Hospital (Zhenjiang First People's Hospital) Zhenjiang Jiangsu China

**Keywords:** acute exacerbation of chronic heart failure, albumin, all‐cause mortality, high‐sensitivity C‐reactive protein, high‐sensitivity C‐reactive protein to albumin ratio

## Abstract

**Objective:**

To evaluate the correlation between the high‐sensitivity C‐reactive protein‐to‐albumin ratio (CAR) and all‐cause mortality in patients with acute exacerbation of chronic heart failure (AECHF).

**Methods:**

In this retrospective cohort study, 342 patients were enrolled from the First People's Hospital of Zhenjiang City between January 1, 2020, and December 31, 2025. Cox regression analyses, subgroup analyses, Kaplan−Meier survival curves, and receiver operating characteristic (ROC) analyses were employed to assess the association between CAR and all‐cause mortality.

**Results:**

During a median follow‐up period of 16.35 months, 95 patients (27.8%) died. Multivariate Cox regression analysis revealed that each unit increase and each standard deviation increase in CAR were associated with a 41.2% and 40.7% increased risk of all‐cause mortality, respectively (HR: 1.412, 95% CI: 1.221−1.633; HR: 1.407, 95% CI: 1.219−1.625). The risk of all‐cause mortality in the high CAR group was 1.778 times that of the low CAR group (HR: 1.778; 95% CI: 1.092−2.894). The robustness of the association between CAR and all‐cause mortality was further corroborated by subgroup and sensitivity analyses (*p* < 0.05). Kaplan–Meier survival curves revealed significant survival differences across CAR subgroups, with markedly lower cumulative survival observed in the high‐CAR group (*p* < 0.05). ROC curve indicated a weak but statistically significant predictive value for all‐cause mortality risk (*p* = 0.025).

**Conclusion:**

Higher levels of CAR are associated with a higher risk of all‐cause mortality in AECHF patients.

## Graphical abstract

In 342 AECHF patients, CAR independently predicted all‐cause mortality. High CAR (>0.66) conferred a 1.778‐fold risk. Despite a modest AUC (0.578), CAR is a practical adjunctive biomarker for multiparametric risk stratification.

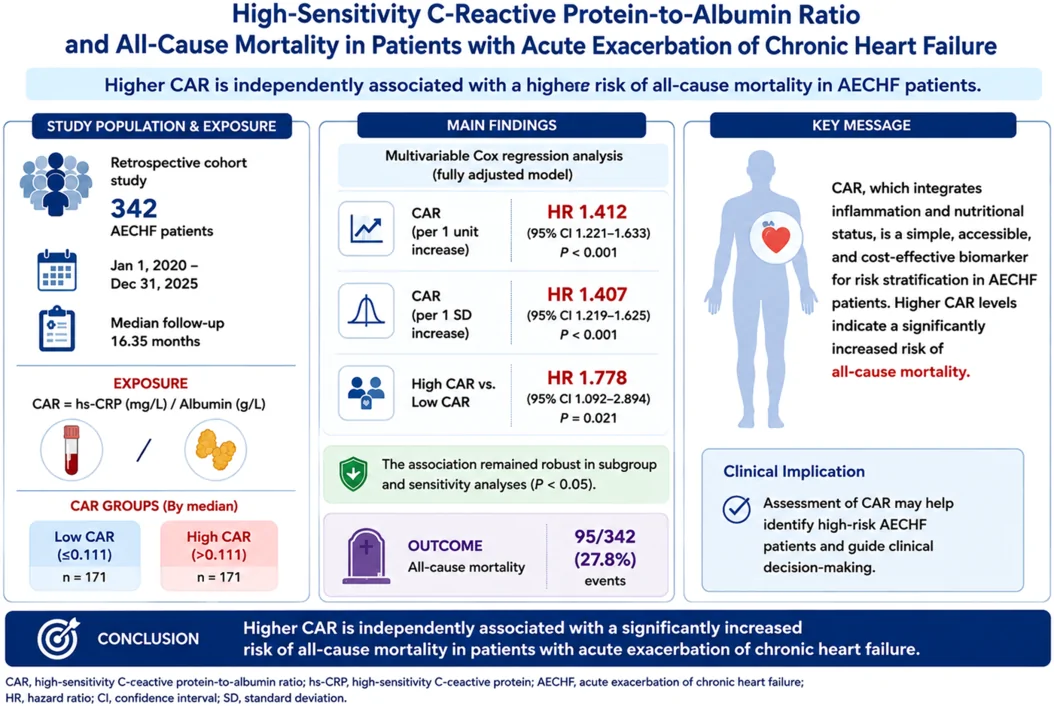

## 1 Introduction

Chronic heart failure (CHF) represents one of the most formidable public health challenges in the 21st‐century cardiovascular landscape. Global epidemiological data indicate that heart failure (HF) affects approximately 64 million people worldwide, with a 5‐year mortality rate remaining alarmingly high at over 50%, imposing an immense burden on healthcare systems globally [1]. Despite significant therapeutic advances in recent years—from the widespread application of angiotensin‐converting enzyme inhibitors (ACEI) and beta‐blockers to angiotensin receptor‐neprilysin inhibitors (ARNI) and sodium‐glucose cotransporter‐2 inhibitors (SGLT2)—the long‐term prognosis of patients with CHF has not been fundamentally improved [2, 3]. Of particular concern, patients experiencing acute exacerbations face substantially elevated risks of rehospitalization and mortality, rendering this clinical subset the most challenging segment in HF management [4]. Therefore, the exploration of reliable, accessible, and cost‐effective prognostic biomarkers for identifying high‐risk individuals and guiding personalized treatment strategies holds urgent clinical significance.

Inflammatory mechanisms play a central role in HF pathogenesis. According to the 2025 ACC Scientific Statement, high‐sensitivity C‐reactive protein (Hs‐CRP) serves as a key marker of residual inflammatory risk [5, 6]. Large‐scale studies have confirmed the long‐term prognostic value of Hs‐CRP. In the ASCOT Legacy Study, Gupta et al. followed 5294 hypertensive patients over 20 years and found that those in the highest Hs‐CRP tertile had a 32% increased risk of non‐fatal myocardial infarction and fatal coronary heart disease (CHD) [7]. Koenig et al. using data from the BiomarCaRE consortium (21 European cohorts, 212 598 individuals), reported that Hs‐CRP ≥ 2 mg/L remained significantly associated with incident CHD even in the absence of traditional risk factors [8]. Meta‐analyses have further consolidated these findings. Yang et al. (28 studies, 60 544 percutaneous coronary intervention [PCI] patients) showed that elevated Hs‐CRP independently predicted cardiovascular mortality and major adverse cardiovascular events (MACE) [9], while Koo et al. reported a nearly twofold increased risk of future cardiovascular events [10]. Collectively, these findings underscore the prognostic significance of Hs‐CRP in cardiovascular disease.

Albumin, the most abundant plasma protein, maintains colloid osmotic pressure and exerts anti‐inflammatory, antioxidant, and transport functions [11]. Hypoalbuminemia is common in HF, driven by hepatic congestion, systemic inflammation, reduced intake, and increased vascular permeability [12, 13]. Epidemiological evidence shows an inverse association between albumin levels and cardiovascular risk, with low albumin corresponding to a 1.5‐fold increased risk [14]. Meta‐analyses in HF confirm hypoalbuminemia as an independent predictor of all‐cause mortality, rehospitalization, and worsening cardiac function [13, 15]. Additionally, prealbumin, a short‐half‐life albumin precursor, is strongly associated with adverse outcomes in chronic HF [16]. Collectively, these findings underscore the prognostic relevance of nutritional markers in HF assessment.

The high‐sensitivity C‐reactive protein‐to‐albumin ratio (CAR) integrates inflammatory burden and nutritional status into a composite marker and has garnered increasing attention in cardiovascular research [17]. CAR captures both pro‐inflammatory pathway activation and catabolic stress, potentially amplifying pathological signals beyond individual biomarkers [18]. Hs‐CRP impairs endothelial function by inhibiting nitric oxide synthesis, while hypoalbuminemia independently affects cardiovascular prognosis irrespective of malnutrition or inflammation [19, 20]. This interaction forms the biological basis for CAR as a composite biomarker. This systematic evaluation establishes, at the evidence‐based medicine level, the independent prognostic value of CAR in HF, providing methodological support for future research.

In recent years, clinical application research on CAR in cardiovascular diseases has deepened, with multiple studies validating its prognostic value from different dimensions. Li et al. demonstrated in a cohort of 2755 patients with type 2 diabetes undergoing PCI that CAR served as an independent predictor of 5‐year all‐cause mortality, and importantly, CAR outperformed Hs‐CRP alone in predictive accuracy [21]. Similarly, Zhang et al. confirmed that CAR, as a novel inflammatory biomarker integrating pro‐inflammatory and nutritional status, provided independent prognostic value for MACE in ST‐segment elevation myocardial infarction (STEMI) patients with hypertension [22]. Notably, this study correlated CAR with classical myocardial injury markers, elucidating from a mechanistic perspective that elevated CAR may reflect the pathophysiological process where increased inflammatory burden and deteriorating nutritional status collectively promote myocardial injury progression.

The predictive value of CAR across different HF phenotypes and special populations is increasingly being elucidated. In a prospective study of 1272 older HF patients (median age 81 years), CAR > 0.240 independently predicted physical frailty (OR: 1.56) and 2‐year all‐cause mortality (HR: 1.44) after multivariable adjustment [23]. In a study of 250 dilated cardiomyopathy patients with HFrEF receiving pacemaker therapy, CAR was an independent predictor of major adverse cardiac events (HR: 4.30), with a cutoff of 0.08 demonstrating good discriminatory ability [24]. These findings underscore the prognostic utility of CAR across elderly and dilated cardiomyopathy HF populations.

Acute exacerbation of CHF (AECHF) represents a critical turning point in the disease trajectory, characterized by pronounced inflammatory activation and rapid nutritional deterioration [25, 26]. The “scissors difference” formed by sharply rising Hs‐CRP and rapidly declining albumin may amplify pathological signals, enabling CAR to more sensitively capture disease dynamics than either single marker [27]. Studies in acute HF have confirmed that admission hs‐CRP levels independently correlate with long‐term mortality and congestion burden [28, 29]. These findings provide a theoretical foundation for applying CAR as a dynamic prognostic marker in the AECHF population.

However, a systematic review of the literature reveals limitations regarding CAR and all‐cause mortality in AECHF. First, study populations are heterogeneous, with most evidence derived from stable HF or outpatient cohorts rather than acute exacerbations [17]. Second, optimal CAR cutoffs vary widely (0.08–2.78), likely due to differences in baseline characteristics, sample size, and outcome definitions [24, 30]. Third, whether CAR provides prognostic value independent of conventional markers such as NT‐proBNP remains controversial, with insufficient evidence stratified by HF phenotypes [31]. Fourth, large‐scale, multicenter data from Chinese AECHF populations are scarce, despite potential racial differences in inflammatory burden and nutritional status [32].

Based on the above research background, in order to address this research gap, this study aims to assess the correlation between CAR and all‐cause mortality in AECHF patients, providing new clues and a theoretical basis for improving the poor prognosis and reducing the mortality rate of AECHF patients.

## 2 Methods

### 2.1 Study Population and Design

This single‐center retrospective cohort study included 342 patients with AECHF admitted to Zhenjiang First People's Hospital from January 1, 2020, to December 31, 2025. Inclusion criteria were: (1) age ≥ 18 years; (2) a definite diagnosis of AECHF according to the 2023 Focused Update of the 2021 ESC Guidelines for the Diagnosis and Treatment of Acute and CHF [33]. Exclusion criteria were: (1) patients receiving anti‐inflammatory or nutritional therapy; (2) patients lacking baseline data on Hs‐CRP or albumin; (3) patients with severe hematological diseases (e.g., lymphoma, leukemia); (4) patients with severe immune system diseases (e.g., rash, systemic lupus erythematosus); (5) patients with severe infectious diseases (e.g., sepsis); (6) patients with severe hepatic or renal failure (e.g., hepatic and renal failure, uremia); (7) patients lost to follow‐up. The study was approved by the Ethics Committee of Zhenjiang First People's Hospital ([2026] KYW001‐82). The research protocol conformed to the principles of the Declaration of Helsinki. All patients provided informed consent.

### 2.2 Variable Collection and Definition

All variables analyzed in this study were collected from the hospital's electronic medical record system, including demographic data, anthropometric measurements, past medical history and comorbidities, blood biomarkers, echocardiographic data, and medications at discharge. Demographic data included age, sex, and smoking (defined as a history of regular smoking with current smoking status). Anthropometric data included height, weight, body mass index (BMI), systolic blood pressure (SBP), and diastolic blood pressure (DBP). BMI was calculated as weight in kilograms divided by height in meters squared (kg/m^2^).

Data on past medical history and comorbidities included diabetes, hypertension, dyslipidemia, atrial fibrillation, CHD, stroke, revascularization, and malignancy. Diabetes was defined as a previous definitive diagnosis, or a fasting blood glucose (FBG) ≥ 7.0 mmol/L or glycated hemoglobin (HbA1c) ≥ 6.5% during this hospitalization [34]. Hypertension was defined as a previous definitive diagnosis, or SBP ≥ 140 mmHg or DBP ≥ 90 mmHg during this hospitalization [35]. Dyslipidemia was defined as total cholesterol ≥ 6.2 mmol/L, low‐density lipoprotein cholesterol (LDL‐C) ≥ 4.1 mmol/L, triglycerides ≥ 2.3 mmol/L, or high‐density lipoprotein cholesterol (HDL‐C) < 1.0 mmol/L during this hospitalization [36]. Atrial fibrillation was defined as a rapid supraventricular arrhythmia characterized on surface electrocardiogram by the replacement of consistent P waves by rapid oscillations or fibrillatory waves (f waves) and an irregularly irregular ventricular response in the absence of atrioventricular block [37]. CHD was defined as myocardial ischemia, hypoxia, or necrosis resulting from luminal narrowing or occlusion of coronary arteries due to atherosclerosis [38]. Revascularization was defined as the restoration of blood supply to ischemic tissues or organs via surgical or interventional procedures [39]. Stroke was defined as a neurological deficit attributed to an acute focal injury of the central nervous system by a vascular cause, including cerebral infarction, intracerebral hemorrhage, and subarachnoid hemorrhage, with clinical symptoms persisting for more than 24 h [40].

Blood biomarkers included fibrinogen, d‐dimer, NT‐proBNP, creatine kinase‐myocardial band (CK‐MB), cardiac troponin I (cTnI), FBG, HbA1c, alanine aminotransferase (ALT), aspartate aminotransferase (AST), total bilirubin, Hs‐CRP, albumin, uric acid, estimated glomerular filtration rate (eGFR), triglycerides, total cholesterol, LDL‐C, HDL‐C, serum potassium, serum sodium, and serum calcium. Hs‐CRP was measured using high‐sensitivity techniques such as immunoturbidimetry, with units reported in milligrams per liter (mg/L). Albumin was measured using the bromocresol green (BCG) colorimetric method, with units reported in grams per deciliter (g/L). Echocardiographic data included LVEF, which was measured using echocardiography and reported as a percentage (%). Medications at discharge included aspirin, clopidogrel, beta‐blockers, ACEI, angiotensin II receptor blockers (ARB), calcium channel blockers (CCB), ARNI, nitrates, and hypoglycemic agents.

### 2.3 Definition and Grouping of CAR

In this study, CAR was defined as the ratio of Hs‐CRP (mg/L) to albumin (g/L). To ensure comparability with other studies, CAR was not only analyzed as a raw continuous variable but also standardized: standardized CAR = (CAR−mean)/standard deviation (SD). Furthermore, based on the 85th percentile of CAR (0.66), patients were divided into two groups: the low CAR group (≤0.66, *n* = 291) and the high CAR group (>0.66, *n* = 51).

### 2.4 Follow‐Up and Outcomes

A total of 389 patients were initially screened, of whom 47 were excluded due to loss to follow‐up, leaving 342 patients for the final analysis. Follow‐up started from the date of hospital discharge and continued until the occurrence of all‐cause death or the study end date (December 31, 2025), whichever came first. Follow‐up was conducted through multiple outpatient and emergency department records, hospitalization data, and telephone interviews. The primary outcome was all‐cause mortality, defined as death from any cause.

### 2.5 Statistical Analysis

All statistical analyses were performed using SPSS version 26.0. The normality of all continuous variables was assessed using the Shapiro–Wilk test prior to analysis. Continuous variables with a normal distribution were expressed as mean ± SD, and differences between two groups were evaluated using the independent sample *t*‐test. Continuous variables with a non‐normal distribution were expressed as median (interquartile range), and differences between two groups were assessed using the nonparametric Mann–Whitney *U* test. Categorical variables were presented as frequencies (percentages), and differences between two groups were compared using the chi‐square test or Fisher's exact test, as appropriate. Univariate Cox regression analysis was used to evaluate the association between each variable and all‐cause mortality. Variables with *p* < 0.05 were then selected for inclusion in three multivariate Cox regression models. Model 1 was adjusted only for age. Model 2 was adjusted for age, BMI, and SBP. Model 3 was adjusted for age, SBP, BMI, uric acid, LDL‐C, NT‐proBNP, aspirin, clopidogrel, beta‐blockers, and ARNI.

The variable selection for the multivariate models followed a stepwise, clinically integrated strategy. Candidate variables were first screened by univariate Cox regression (*p* < 0.05), but final inclusion was further refined based on established clinical relevance. Specifically, Model 1 and Model 2 served as progressive adjustments for core demographic and hemodynamic factors (age, BMI, and SBP). Model 3 was expanded to incorporate additional established cardiovascular risk factors (uric acid, LDL‐C, NT‐proBNP) and key discharge medications (aspirin, clopidogrel, beta‐blockers, ARNI) to minimize residual confounding from disease severity and evidence‐based therapies. To strictly control the risk of overfitting given 95 mortality events, we adhered to the Events Per Variable (EPV) principle and limited Model 3 to a maximum of 10 covariates. Other clinically relevant confounders (e.g., eGFR) were not forced into the main models to preserve parsimony, but were instead evaluated in separate sensitivity analyses.

Subgroup analyses were conducted to further evaluate the association between CAR and all‐cause mortality across different subgroups based on age, sex, smoking, diabetes, hypertension, dyslipidemia, atrial fibrillation, and CHD. The difference in cumulative survival between different CAR groups was assessed using Kaplan–Meier survival curves, with differences compared by the log‐rank test. Finally, the predictive value of CAR for all‐cause mortality was evaluated using receiver operating characteristic (ROC) curve analysis, with the area under the curve (AUC) and its 95% confidence interval (CI) reported. All tests were two‐tailed, and a *p* value < 0.05 was considered statistically significant.

## 3 Results

### 3.1 Baseline Characteristics Grouped by CAR

As shown in Table 1, based on the 85th percentile of CAR (0.66): the low CAR group (≤0.66, *n* = 291) and the high CAR group (>0.66, *n* = 51). Compared with the low CAR group, the high CAR group had higher rates of malignancy, as well as higher heart rate, fibrinogen, d‐dimer, hs‐CRP, and cTnI, along with lower levels of albumin, HDL‐C, sodium, calcium, and reduced use of nitrates (all *p* < 0.05). However, no significant differences were observed in other variables between the two groups (*p* > 0.05).

**Table 1:** Baseline characteristics grouped by CAR.

Variables	Total population	Low CAR (≤0.66)	High CAR (>0.66)	*p* value
*N*	342	291	51	
Age, years	76.50 (68.00, 82.00)	77.00 (68.00, 81.00)	76.00 (68.00, 84.00)	0.930
*Sex, n (%)*				0.425
Male	204 (59.6%)	171 (58.8%)	33 (64.7%)	
Female	138 (40.4%)	120 (41.2%)	18 (35.3%)	
Smoking, *n* (%)	117 (34.20%)	95 (32.6%)	22 (43.1%)	0.145
Diabetes, *n* (%)	154 (45.0%)	129 (44.3%)	25 (49.0%)	0.535
Hypertension, *n* (%)	225 (65.8%)	194 (66.7%)	31 (60.8%)	0.414
Dyslipidemia, *n* (%)	116 (33.9%)	95 (32.6%)	21 (41.2%)	0.235
Atrial fibrillation, *n* (%)	180 (52.6%)	155 (53.3%)	25 (49.0%)	0.575
Coronary heart disease, *n* (%)	194 (56.7%)	168 (57.7%)	26 (51.0%)	0.369
Stroke, *n* (%)	63 (18.4%)	52 (17.9%)	11 (21.6%)	0.530
Revascularization, *n* (%)	49 (14.3%)	39 (13.4%)	10 (19.6%)	0.243
Malignancy, *n* (%)	31 (9.1%)	22 (7.6%)	9 (17.6%)	0.032
NYHA functional class (III‐IV), *n* (%)	318 (93.0%)	269 (92.4%)	49 (96.1%)	0.552
BMI, kg/m^2^	23.20 (20.80, 24.80)	23.20 (20.80, 24.80)	23.20 (22.20, 25.40)	0.422
SBP, mmHg	126.00 (111.80, 142.00)	126.00 (112.00, 143.00)	124.00 (106.00, 137.00)	0.217
DBP, mmHg	78.50 (69.00, 91.00)	79.00 (70.00, 91.00)	75.00 (65.00, 91.00)	0.163
HR, beats/min	79.00 (69.00, 92.00)	78.00 (69.00, 91.00)	81.60 (72.00, 98.00)	0.045
Fibrinogen, g/L	3.10 (2.50, 4.60)	2.97 (2.44, 3.48)	4.11 (3.17, 5.38)	< 0.001
d‐dimer, mg/L	1.29 (0.74, 2.44)	1.22 (0.72, 2.32)	2.29 (0.90, 3.05)	< 0.001
Nt‐ProBNP, pg/mL	6378.92 (2606.13, 9379.72)	6076.00 (2603.75, 9049.00)	6995.67 (2829.97, 16216.00)	0.114
CK‐MB, U/L	8.55 (5.10, 12.45)	8.60 (5.10, 12.40)	7.80 (5.20, 12.80)	0.924
cTnI, ng/mL	0.02 (0.02, 0.02)	0.02 (0.02, 0.02)	0.02 (0.02, 0.22)	0.014
FBG, mmol/L	5.22 (4.48, 6.64)	5.18 (4.50, 6.43)	5.62 (4.38, 8.95)	0.127
HbA1c, %	6.40 (5.80, 6.83)	6.30 (5.80, 6.80)	6.65 (5.80, 7.00)	0.535
ALT, U/L	19.00 (12.00, 34.00)	20.00 (13.00, 34.00)	15.00 (10.00, 30.00)	0.127
AST, U/L	23.00 (18.00, 34.00)	23.00 (18.00, 33.00)	25.00 (14.00, 40.00)	0.882
Total bilirubin, µmol/L	18.20 (13.38, 24.80)	18.10 (14.00, 24.80)	19.20 (9.90, 25.30)	0.190
Hs‐CRP, mg/L	4.87 (1.74, 11.82)	3.72 (1.46, 7.99)	36.89 (26.36, 79.20)	< 0.001
ALB, g/L	34.55 (31.98, 37.10)	35.00 (33.20, 37.40)	30.60 (28.10, 33.70)	< 0.001
CAR	0.14 (0.05, 0.38)	0.11 (0.04, 0.23)	1.31 (0.81, 2.56)	< 0.001
Uric acid, µmol/L	438.50 (341.00, 543.25)	431.00 (341.00, 525.00)	474.00 (337.00, 619.00)	0.085
eGFR, mL/min/1.73 m^2^	59.71 (42.12, 78.36)	60.09 (2.13, 78.64)	55.21 (40.31, 74.76)	0.243
Triglycerides, mmol/L	0.96 (0.72, 1.24)	0.96 (0.71, 1.26)	0.94 (0.76, 1.09)	0.933
Total cholesterol, mmol/L	3.45 (2.79, 4.24)	3.47 (2.80, 4.29)	3.35 (2.75, 3.88)	0.618
LDL‐C, mmol/L	1.95 (1.46, 2.47)	1.93 (1.46, 2.52)	2.04 (1.52, 2.27)	0.988
HDL‐C, mmol/L	1.17 (0.96, 1.43)	1.18 (0.97, 1.43)	1.10 (0.88, 1.29)	0.049
K^+^, mmol/L	3.96 (3.59, 4.36)	3.98 (3.61, 4.36)	3.92 (3.44, 4.29)	0.238
Na^+^, mmol/L	139.45 (137.35, 141.50)	139.80 (138.10, 141.60)	136.80 (132.10, 140.40)	< 0.001
Ca^2+^, mmol/L	2.18 (2.09, 2.25)	2.19 (2.10, 2.26)	2.13 (2.02, 2.20)	< 0.001
LVEF, %	39.00 (28.00, 53.25)	38.00 (28.00, 53.00)	43.00 (27.00, 59.00)	0.546
*Discharge medication, n (%)*				
Aspirin	134 (39.2%)	116 (39.9%)	18 (35.3%)	0.538
Clopidogrel	81 (23.7%)	69 (23.7%)	12 (23.5%)	0.978
Statin	254 (74.3%)	220 (75.6%)	34 (66.7%)	0.178
Beta blocker	241 (70.5%)	209 (71.8%)	32 (62.7%)	0.190
ACEI/ARB	70 (20.5%)	61 (21.0%)	9 (17.6%)	0.588
CCB	60 (17.5%)	53 (18.2%)	7 (13.7%)	0.437
ARNI	186 (54.4%)	162 (55.7%)	24 (47.1%)	0.255
Nitrates	89 (26.0%)	70 (24.1%)	19 (37.3%)	0.048
Antidiabetic drugs	112 (32.7%)	94 (32.3%)	18 (35.3%)	0.675
*All‐cause mortality, n (%)*				0.008
Yes	95 (27.8%)	73 (25.1%)	22 (43.1%)	
No	247 (72.2%)	218 (74.9%)	29 (56.9%)	

Abbreviations: ACEI, angiotensin converting enzyme inhibitor; ALB, albumin; ALT, alanine aminotransferase; ARB, angiotensin II receptor blockers; ARNI, angiotensin receptor‐neprilysin inhibitor; AST, aspartate aminotransferase; BMI, body mass index; CAR, C‐reactive protein to albumin ratio; CCB, calcium channel blockers; CK‐MB, creatine kinase MB form; cTnI, cardiac troponin I; DBP, diastolic blood pressure; eGFR, estimated glomerular filtration rate; FBG, fasting blood glucose; HbA1c, glycosylated hemoglobin A1c; HDL‐C, high‐density lipoprotein cholesterol; HR, heart rate; Hs‐CRP, high‐sensitivity C‐reactive protein; NT‐proBNP, N‐terminal pro‐B‐type natriuretic peptide; NYHA functional class, New York Heart Association functional class; LDL‐C, low‐density lipoprotein cholesterol; LVEF, left ventricular ejection fractions; SBP, systolic blood pressure.

During a median follow‐up period of 16.35 months, a total of 95 patients (27.8%) reached the primary endpoint of all‐cause mortality, while the remaining 247 patients (72.2%) were censored, including those who survived until the study cutoff date and those who were lost to follow‐up during the observation period. And Table 1 showed that, compared with the low CAR group, the all‐cause mortality rate in the high CAR group was higher (*p* = 0.008).

### 3.2 Baseline Characteristics According to All‐Cause Mortality Groups

As shown in Table 2, patients were divided into two groups based on all‐cause mortality: the non‐all‐cause mortality group (*n* = 247) and the all‐cause mortality group (*n* = 95). Compared with the non‐all‐cause mortality group, the all‐cause mortality group exhibited significantly higher age, d‐dimer, NT‐proBNP, and uric acid levels, along with lower BMI, SBP, DBP, ALT, albumin, eGFR, triglycerides, total bilirubin, LDL‐C, sodium, calcium, as well as reduced use of aspirin, clopidogrel, beta‐blockers, and ARNI (all *p* < 0.05). However, no statistically significant differences were observed in other variables between the two groups (*p* > 0.05).

**Table 2:** Baseline characteristics according to all‐cause mortality groups.

Variables	Non‐all‐cause mortality	All‐cause mortality	*p* value
*N*	247	95	
Age, years	75.00 (68.00, 80.00)	79.00 (71.00, 86.00)	< 0.001
Sex, *n* (%)			0.682
Male	149 (60.3%)	55 (57.9%)	
Female	98 (39.7%)	40 (42.1%)	
Smoking, *n* (%)	84 (34.0%)	33 (34.7%)	0.899
Diabetes, *n* (%)	109 (44.1%)	45 (47.4%)	0.590
Hypertension, *n* (%)	158 (64.0%)	67 (70.5%)	0.252
Dyslipidemia, *n* (%)	83 (33.6%)	33 (34.7%)	0.843
Atrial fibrillation, *n* (%)	129 (52.2%)	51 (53.7%)	0.809
Coronary heart disease, *n* (%)	142 (57.5%)	52 (54.7%)	0.645
Stroke, *n* (%)	44 (17.8%)	19 (20.0%)	0.640
Revascularization, *n* (%)	37 (15.0%)	12 (12.6%)	0.579
Malignancy, *n* (%)	21 (8.5%)	10 (10.5%)	0.559
NYHA functional class (III‐IV), *n* (%)	226 (91.5%)	92 (96.8%)	0.083
BMI, kg/m^2^	23.31 (21.63, 25.32)	23.05 (19.69, 23.52)	0.001
SBP, mmHg	128.00 (114.00, 147.00)	116.00 (102.00, 135.00)	< 0.001
DBP, mmHg	82.00 (72.00, 94.00)	71.00 (63.00, 83.00)	< 0.001
HR, beats/min	80.00 (70.00, 92.00)	76.00 (67.00, 91.00)	0.091
Fibrinogen, g/L	3.12 (2.53, 3.65)	2.91 (2.32, 3.62)	0.194
d‐dimer, mg/L	1.22 (0.70, 2.32)	1.45 (0.78, 2.67)	0.025
Nt‐ProBNP, pg/mL	5505.00 (2001.04, 8783.86)	8783.86 (3238.53, 13920.00)	0.001
CK‐MB, U/L	8.40 (4.97, 12.30)	8.60 (5.32, 12.90)	0.373
cTnI, ng/mL	0.02 (0.02, 0.02)	0.02 (0.02, 0.05)	0.323
FBG, mmol/L	5.18 (4.48, 6.45)	5.25 (4.48, 6.85)	0.529
HbA1c, %	6.40 (5.80, 6.80)	6.40 (5.80, 7.00)	0.875
ALT, U/L	20.00 (13.00, 35.00)	15.00 (10.00, 30.00)	0.010
AST, U/L	23.00 (18.00, 33.00)	23.00 (17.00, 37.00)	0.831
Total bilirubin, µmol/L	18.20 (13.50, 24.20)	17.90 (12.90, 27.80)	0.595
Hs‐CRP, mg/L	4.25 (1.62, 11.16)	5.71 (2.14, 18.83)	0.058
ALB, g/L	35.10 (33.20, 37.60)	33.00 (30.40, 34.80)	< 0.001
CAR	0.13 (0.05, 0.34)	0.17 (0.06, 0.55)	0.025
Uric acid, µmol/L	421.00 (337.00, 511.00)	495.00 (377.00, 600.00)	< 0.001
eGFR, mL/min/1.73 m^2^	61.89 (46.20, 80.36)	46.16 (32.39, 69.10)	< 0.001
Triglycerides, mmol/L	1.01 (0.76, 1.30)	0.78 (0.58, 1.12)	< 0.001
Total cholesterol, mmol/L	3.56 (2.90, 4.42)	3.25 (2.67, 3.78)	0.005
LDL‐C, mmol/L	2.03 (1.52, 2.65)	1.83 (1.32, 2.21)	0.011
HDL‐C, mmol/L	1.17 (0.97, 1.43)	1.18 (0.95, 1.43)	0.681
K^+^, mmol/L	3.94 (3.61, 4.35)	4.02 (3.55, 4.41)	0.739
Na^+^, mmol/L	139.85 (137.70, 141.60)	139.00 (135.90, 141.30)	0.009
Ca^2+^, mmol/L	2.19 (2.10, 2.26)	2.15 (2.06, 2.24)	0.014
LVEF, %	39.00 (28.00, 52.00)	40.86 (27.00, 61.00)	0.419
Discharge medication, *n* (%)			
Aspirin	106 (42.9%)	28 (29.5%)	0.023
Clopidogrel	67 (27.1%)	14 (14.7%)	0.016
Statin	188 (76.1%)	66 (69.5%)	0.208
Beta blocker	183 (74.1%)	58 (61.1%)	0.018
ACEI/ARB	52 (21.1%)	18 (18.9%)	0.666
CCB	44 (17.8%)	16 (16.8%)	0.832
ARNI	147 (59.5%)	39 (41.1%)	0.002
Nitrates	63 (25.5%)	26 (27.4%)	0.725
Antidiabetic drugs	80 (32.4%)	32 (33.7%)	0.819

Abbreviations: ACEI, angiotensin converting enzyme inhibitor; ALB, albumin; ALT, alanine aminotransferase; ARB, angiotensin II receptor blockers; ARNI, angiotensin receptor‐neprilysin inhibitor; AST, aspartate aminotransferase; BMI, body mass index; CAR, C‐reactive protein to albumin ratio; CCB, calcium channel blockers; CK‐MB, creatine kinase MB form; cTnI, cardiac troponin I; DBP, diastolic blood pressure; eGFR, estimated glomerular filtration rate; FBG, fasting blood glucose; HbA1c, glycosylated hemoglobin A1c; HDL‐C, high‐density lipoprotein cholesterol; Hs‐CRP, high‐sensitivity C‐reactive protein; LDL‐C, low‐density lipoprotein cholesterol; LVEF, left ventricular ejection fractions; NT‐proBNP, N‐terminal pro‐B‐type natriuretic peptide; NYHA functional class, New York Heart Association functional class; SBP, systolic blood pressure.

### 3.3 Univariate Cox Regression Analysis of All‐Cause Mortality

As shown in Table 3, univariate Cox regression analysis revealed that age, BMI, SBP, DBP, d‐dimer, NT‐proBNP, total bilirubin, Hs‐CRP, ALB, UA, eGFR, triglycerides, total cholesterol, LDL‐C, sodium, aspirin, clopidogrel, beta‐blockers, and ARNI were significantly associated with the risk of all‐cause mortality (*p* < 0.05). However, no significant association was observed between other variables and the risk of all‐cause mortality (*p* > 0.05). Specifically, for each unit increase in CAR, the risk of all‐cause mortality increased by 37.7% (HR 1.377, 95% CI: 1.199−1.581).

**Table 3:** Univariate Cox regression analysis of all‐cause mortality.

Variables	HR	95% CI	*p* value
Age	1.034	1.013, 1.056	0.002
Male	0.910	0.605, 1.368	0.650
Smoking	1.004	0.658, 1.533	0.984
Diabetes	1.082	0.723, 1.620	0.701
Hypertension	1.312	0.844, 2.040	0.227
Dyslipidemia	1.033	0.677, 1.575	0.882
Atrial fibrillation	1.015	0.678, 1.519	0.944
Coronary heart disease	0.898	0.599, 1.345	0.601
Stroke	1.242	0.750, 2.057	0.400
Revascularization	0.833	0.455, 1.527	0.555
Malignancy	1.233	0.640, 2.375	0.532
NYHA functional class (III−IV), *n* (%)	2.544	0.806, 8.036	0.111
BMI	0.908	0.858, 0.961	0.001
SBP	0.983	0.973, 0.992	< 0.001
DBP	0.962	0.947, 0.977	< 0.001
HR	0.993	0.982, 1.005	0.270
Fibrinogen	0.922	0.743, 1.143	0.459
d‐dimer	1.091	1.002, 1.187	0.044
Nt‐ProBNP	1.000	1.000, 1.000	0.001
CK‐MB	1.002	0.985, 1.018	0.838
cTnI	0.960	0.767, 1.202	0.723
FBG	1.067	0.993, 1.147	0.076
HbA1c	0.970	0.823, 1.143	0.719
ALT	0.998	0.991, 1.005	0.527
AST	1.000	0.997, 1.002	0.696
Total bilirubin	1.022	1.007, 1.037	0.003
Hs‐CRP	1.011	1.005, 1.016	< 0.001
ALB	0.931	0.899, 0.964	< 0.001
CAR	1.377	1.199, 1.581	< 0.001
Uric acid	1.002	1.001, 1.004	< 0.001
eGFR	0.980	0.971, 0.989	< 0.001
Triglycerides	0.408	0.243, 0.686	0.001
Total cholesterol	0.771	0.629, 0.944	0.012
LDL‐C	0.701	0.527, 0.933	0.015
HDL‐C	0.933	0.545, 1.596	0.800
K^+^	1.170	0.820, 1.668	0.387
Na^+^	0.922	0.884, 0.961	< 0.001
Ca^2+^	0.579	0.196, 1.709	0.322
LVEF	1.007	0.944, 1.020	0.292
Discharge medication			
Aspirin	0.613	0.394, 0.953	0.030
Clopidogrel	0.508	0.288, 0.895	0.019
Statin	0.735	0.475, 1.138	0.167
Beta blocker	0.642	0.425, 0.969	0.035
ACEI/ARB	0.760	0.453, 1.275	0.298
CCB	0.877	0.512, 1.502	0.633
ARNI	0.564	0.375, 0.849	0.006
Nitrates	1.048	0.667, 1.645	0.840
Antidiabetic drugs	1.064	0.695, 1.629	0.775

Abbreviations: ACEI, angiotensin converting enzyme inhibitor; ALB, albumin; ALT, alanine aminotransferase; ARB, angiotensin II receptor blockers; ARNI, angiotensin receptor‐neprilysin inhibitor; AST, aspartate aminotransferase; BMI, body mass index; CAR, C‐reactive protein to albumin ratio; CCB, calcium channel blockers; CI, confidence interval; CK‐MB, creatine kinase MB form; cTnI, cardiac troponin I; DBP, diastolic blood pressure; eGFR, estimated glomerular filtration rate; FBG, fasting blood glucose; HbA1c, glycosylated hemoglobin A1c; HDL‐C, high‐density lipoprotein cholesterol; HR, heart rate; HR, hazard ratio; Hs‐CRP, high‐sensitivity C‐reactive protein; LDL‐C, low‐density lipoprotein cholesterol; LVEF, left ventricular ejection fractions; NT‐proBNP, N‐terminal pro‐B‐type natriuretic peptide; NYHA functional class, New York Heart Association functional class; SBP, systolic blood pressure.

### 3.4 Multivariate Cox Regression Analysis of All‐Cause Mortality

As shown in Table 4, multivariate Cox regression analysis revealed the following results: In Model 1 (adjusted for age only), per 1‐unit and per 1‐SD increment in CAR were associated with 38.1% and 37.6% increases in the risk of all‐cause mortality, respectively (HR 1.381, 95% CI: 1.207–1.580; HR 1.376, 95% CI: 1.204–1.573). The risk of all‐cause mortality in the high CAR group was 2.072‑fold higher than that in the low CAR group (HR 2.072, 95% CI: 1.283–3.344). In Model 2 (adjusted for age, BMI and SBP), per 1‐unit and per 1‐SD increment in CAR were associated with 45.7% and 45.1% increases in the risk of all‐cause mortality, respectively (HR 1.457, 95% CI: 1.265–1.677; HR 1.451, 95% CI: 1.262–1.669). The risk of all‐cause mortality in the high CAR group was 1.933‑fold higher than that in the low CAR group (HR 1.933, 95% CI: 1.196–3.123). In Model 3 (further adjusted for age, BMI, SBP, uric acid, LDL‑C, Nt‑proBNP, aspirin, clopidogrel, beta‑blockers and ARNI), per 1‐unit and per 1‐SD increment in CAR were associated with 41.2% and 40.7% increases in the risk of all‐cause mortality, respectively (HR 1.412, 95% CI: 1.221–1.633; HR 1.407, 95% CI: 1.219–1.625). The risk of all‐cause mortality in the high CAR group was 1.778‑fold higher than that in the low CAR group (HR 1.778, 95% CI: 1.092–2.894).

**Table 4:** Multivariate Cox regression analysis of all‐cause mortality.

Variables	Model 1		Model 2		Model 3	
	HR (95% CI)	*p* value	HR (95% CI)	*p* value	HR (95% CI)	*p* value
*CAR as a continuous variable*						
For every 1 unit increase	1.381 (1.207, 1.580)	< 0.001	1.457 (1.265, 1.677)	< 0.001	1.412 (1.221, 1.633)	< 0.001
For every 1 standard deviation increase	1.376 (1.204, 1.573)	< 0.001	1.451 (1.262, 1.669)	< 0.001	1.407 (1.219, 1.625)	< 0.001
CAR as a categorical variable						
Low CAR	Ref		Ref		Ref	
High CAR	2.072 (1.283, 3.344)	0.003	1.933 (1.196, 3.123)	0.007	1.778 (1.092, 2.894)	0.021

*Note:* Model 1: adjusted for age only; Model 2: adjusted for age, BMI, and SBP; Model 3: adjusted for age, BMI, SBP, UA, LDL‐C, Nt‐ProBNP, aspirin, clopidogrel, beta blocker, and ARNI.

Abbreviations: ARNI, angiotensin receptor‐neprilysin inhibitor; BMI, body mass index; CI, confidence interval; CAR, C‐reactive protein to albumin ratio; HR, hazard ratio; LDL‐C, low‐density lipoprotein cholesterol; NT‐proBNP, N‐terminal pro‐B‐type natriuretic peptide; Ref, reference; SBP, systolic blood pressure; UA, uric acid.

### 3.5 Multivariable Subgroup Analysis of All‐Cause Mortality

As shown in Table 5, multivariable subgroup regression analysis revealed that after adjusting for all confounders, in the subgroup aged ≤ 75 years, each unit increase and each SD increase in CAR were associated with a 53.9% and 53.2% higher risk of all‐cause mortality, respectively (*p* < 0.05); in the subgroup aged > 75 years, each unit increase and each SD increase in CAR were associated with a 46.4% and 45.8% higher risk of all‐cause mortality, respectively, and the high CAR group had a 1.892‐fold higher risk of all‐cause mortality compared with the low CAR group (*p* < 0.05). In the female subgroup, each unit increase and each SD increase in CAR were associated with a 51.8% and 51.2% higher risk of all‐cause mortality, respectively, and the high CAR group had a 3.963‐fold higher risk of all‐cause mortality compared with the low CAR group (*p* < 0.05).

**Table 5:** Multivariable subgroup analysis of all‐cause mortality.

Variables	CAR		Standardization CAR		CAR (High vs Low)		
	HR (95% CI)	*p* value	HR (95% CI)	*p* value	HR (95% CI)	*p* value	P for interaction
*Age*							0.799
≤75 years	1.539 (1.129, 2.097)	0.006	1.532 (1.128, 2.082)	0.006	2.094 (0.736, 5.959)	0.166	
>75 years	1.464 (1.243, 1.724)	< 0.001	1.458 (1.240, 1.714)	< 0.001	1.892 (1.068, 3.354)	0.029	
*Sex*							0.382
Male	1.218 (0.896, 1.654)	0.208	1.215 (0.897, 1.646)	0.208	1.009 (0.491, 2.072)	0.981	
Female	1.518 (1.276, 1.807)	< 0.001	1.512 (1.273, 1.796)	< 0.001	3.963 (1.873, 8.387)	< 0.001	
*Smoking*							0.638
Yes	1.222 (0.852, 1.753)	0.275	1.220 (0.854, 1.743)	0.275	1.111 (0.450, 2.741)	0.820	
No	1.438 (1.217, 1.700)	< 0.001	1.433 (1.215, 1.691)	< 0.001	2.047 (1.108, 3.783)	0.022	
*Diabetes*							0.986
Yes	1.422 (1.185, 1.706)	< 0.001	1.417 (1.183, 1.697)	< 0.001	3.348 (1.605, 6.982)	0.001	
No	1.560 (1.226, 1.984)	< 0.001	1.553 (1.223, 1.971)	< 0.001	0.978 (0.434, 2.202)	0.956	
*Hypertension*							0.248
Yes	1.377 (1.136, 1.670)	0.001	1.373 (1.135, 1.661)	0.001	1.484 (0.761, 2.893)	0.246	
No	1.739 (1.360, 2.223)	< 0.001	1.729 (1.356, 2.205)	< 0.001	3.473 (1.551, 7.777)	0.002	
*Dyslipidemia*							0.150
Yes	1.182 (0.768, 1.820)	0.446	1.180 (0.770, 1.809)	0.446	0.578 (0.197, 1.696)	0.318	
No	1.431 (1.217, 1.683)	< 0.001	1.426 (1.214, 1.674)	< 0.001	2.120 (1.147, 3.921)	0.017	
*Atrial fibrillation*							0.022
Yes	1.805 (1.476, 2.207)	< 0.001	1.794 (1.471, 2.189)	< 0.001	1.630 (0.792, 3.353)	0.184	
No	1.128 (0.854, 1.490)	0.395	1.127 (0.856, 1.484)	0.395	1.393 (0.625, 3.107)	0.417	
*Coronary heart disease*							0.771
Yes	1.104 (0.762, 1.602)	0.601	1.103 (0.764, 1.594)	0.601	1.817 (0.866, 3.812)	0.114	
No	1.623 (1.355, 1.945)	< 0.001	1.616 (1.351, 1.932)	< 0.001	1.313 (0.606, 2.843)	0.490	

*Note:* Subgroup analysis adjusted for age, BMI, SBP, UA, LDL‐C, Nt‐ProBNP, aspirin, clopidogrel, beta blocker, and ARNI.

Abbreviations: ARNI, angiotensin receptor‐neprilysin inhibitor; BMI, body mass index; CAR, C‐reactive protein to albumin ratio; CI, confidence interval; HR, hazard ratio; LDL‐C, low‐density lipoprotein cholesterol; NT‐proBNP, N‐terminal pro‐B‐type natriuretic peptide; SBP, systolic blood pressure; UA, uric acid.

In the non‐smoking subgroup, each unit increase and each SD increase in CAR were associated with a 43.8% and 43.3% higher risk of all‐cause mortality, respectively, and the high CAR group had a 2.047‐fold higher risk of all‐cause mortality compared with the low CAR group (*p* < 0.05). In the diabetes subgroup, each unit increase and each SD increase in CAR were associated with a 42.2% and 41.7% higher risk of all‐cause mortality, respectively, and the high CAR group had a 3.348‐fold higher risk of all‐cause mortality compared with the low CAR group (*p* < 0.05). In the non‐diabetes subgroup, each unit increase and each SD increase in CAR were associated with a 56.0% and 53.3% higher risk of all‐cause mortality, respectively (*p* < 0.05).

In the hypertension subgroup, each unit increase and each SD increase in CAR were associated with a 37.7% and 37.3% higher risk of all‐cause mortality, respectively; in the non‐hypertension subgroup, each unit increase and each SD increase in CAR were associated with a 73.9% and 72.9% higher risk of all‐cause mortality, respectively, and the high CAR group had a 3.473‐fold higher risk of all‐cause mortality compared with the low CAR group (*p* < 0.05). In the non‐dyslipidemia subgroup, each unit increase and each SD increase in CAR were associated with a 43.1% and 42.6% higher risk of all‐cause mortality, respectively, and the high CAR group had a 2.120‐fold higher risk of all‐cause mortality compared with the low CAR group (*p* < 0.05). In the AF subgroup, each unit increase and each SD increase in CAR were associated with an 80.5% and 79.4% higher risk of all‐cause mortality, respectively (*p* < 0.05). In the non‐CHD subgroup, each unit increase and each SD increase in CAR were associated with a 62.3% and 61.6% higher risk of all‐cause mortality, respectively (*p* < 0.05).

Formal interaction tests (P for interaction) were performed to evaluate whether the prognostic effect of CAR differed across subgroups. No significant interactions were observed for age, sex, smoking, diabetes, hypertension, dyslipidemia, or CHD (all P for interaction > 0.05), indicating that the association between CAR and all‐cause mortality was broadly consistent across these strata. However, a statistically significant interaction was observed for atrial fibrillation (P for interaction = 0.022), suggesting that the predictive value of CAR may be more pronounced in patients with atrial fibrillation compared with those without.

### 3.6 Differences in Cumulative Survival between the Two CAR Subgroups

**Figure 1:**
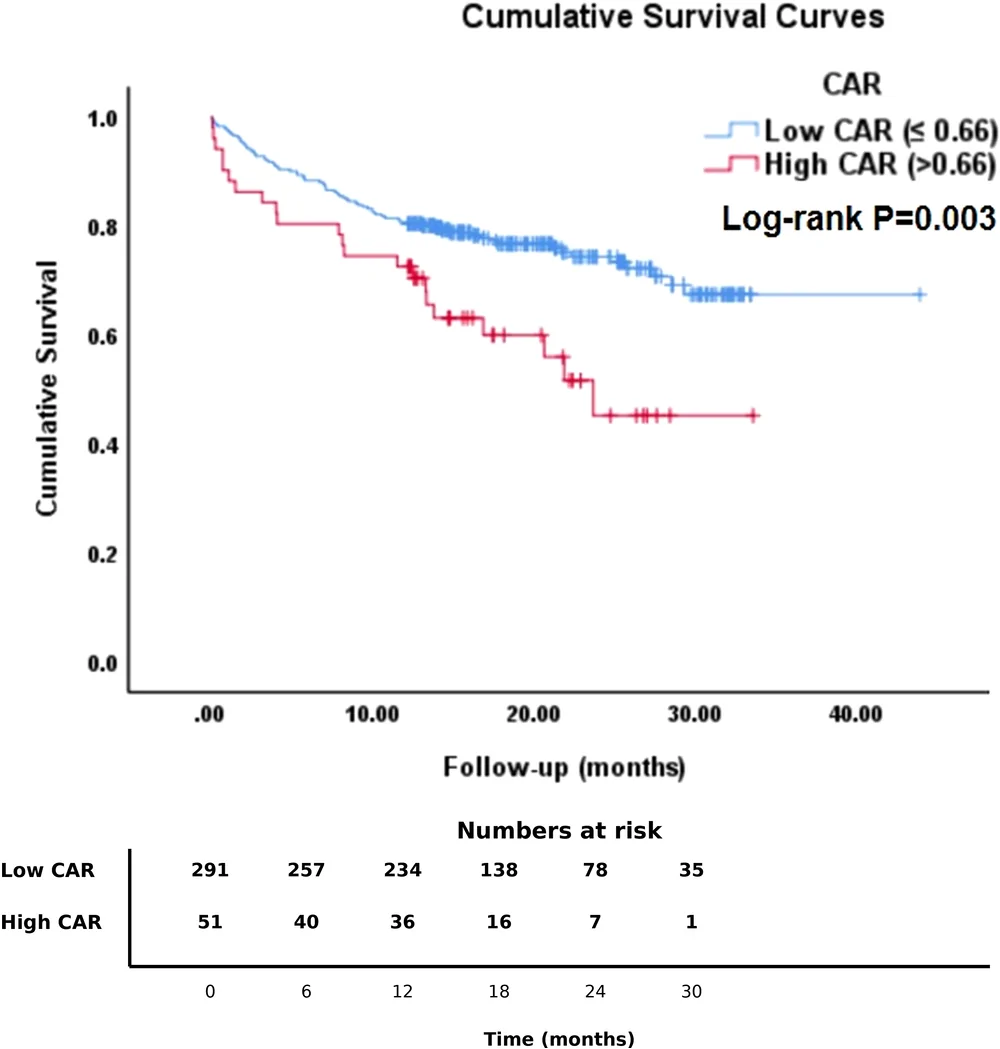
The Kaplan−Meier survival curves for all‐cause mortality between the two groups with different CAR. CAR, C‐reactive protein to albumin ratio.

As shown in Figure 1, Kaplan–Meier survival curves revealed a significant difference in cumulative survival between the two CAR subgroups (Log‐rank *p* = 0.003). Specifically, patients in the high‐CAR group exhibited a markedly lower cumulative survival rate relative to those in the low‐CAR group, indicating an inferior prognosis.

### 3.7 ROC Curve of the Correlation Between CAR and the Risk of All‐Cause Mortality

**Figure 2:**
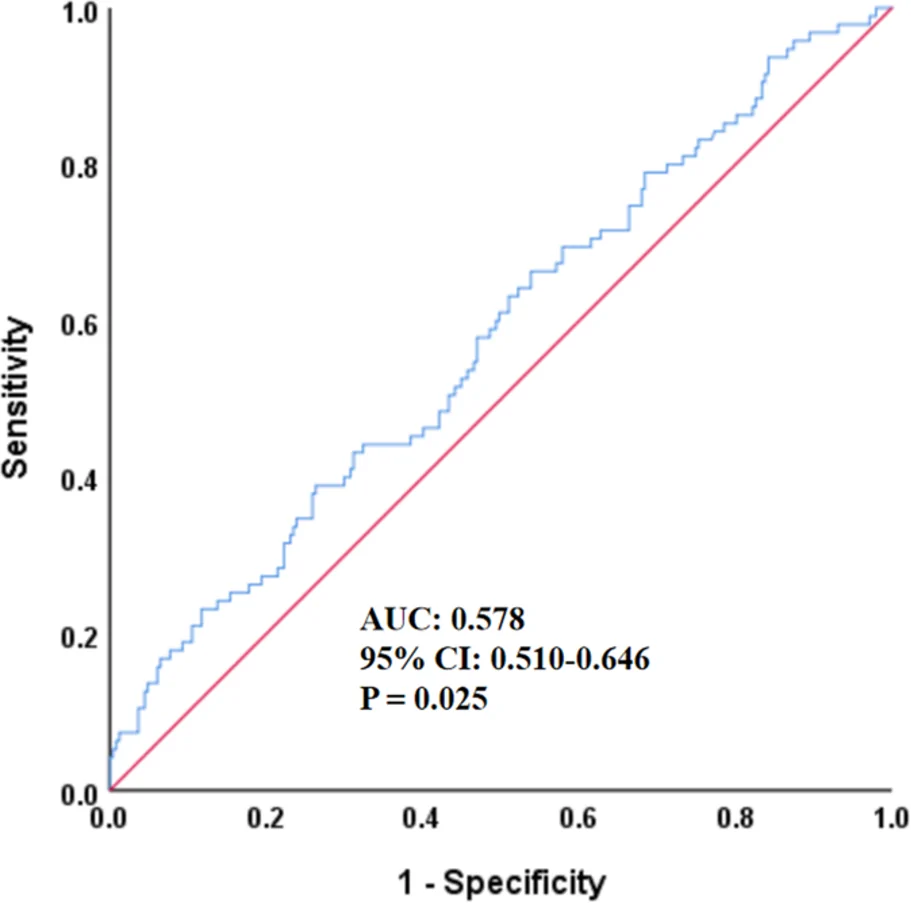
ROC curve of the correlation between CAR and the risk of all‐cause mortality. AUC, area under the curve; CAR, C‐reactive protein to albumin ratio; CI, confidence interval; ROC, receiver operating characteristic.

As shown in Figure 2, ROC curve analysis showed that CAR had a significant but modest predictive value for all‐cause mortality (AUC 0.578, 95% CI: 0.510−0.646, *p* = 0.025).

### 3.8 Sensitivity Analysis Adjusting for eGFR

To verify the robustness of the primary findings, a sensitivity analysis was performed by additionally adjusting for eGFR on the basis of the fully adjusted Model 3. As presented in Table 6, continuous CAR remained an independent predictor of all‐cause mortality after further eGFR adjustment. Each one‐unit increment in CAR was associated with a 40.9% increased risk of all‐cause mortality (HR = 1.409, 95% CI: 1.216–1.632, *p* < 0.001), and each one‐SD increase yielded similar results (HR = 1.404, 95% CI: 1.213–1.624, *p* < 0.001), further consolidating the robustness of our main conclusions. Notably, when CAR was dichotomized into categorical variables, the elevated mortality risk in the high‐CAR group failed to reach statistical significance (HR = 1.418, 95% CI: 0.852–2.361, *p* = 0.179), although a consistent trend remained. This marginal attenuation is likely attributable to reduced statistical power following categorical transformation of continuous exposure variables. Collectively, the association between CAR and all‐cause mortality remained robust after additional correction for eGFR.

**Table 6:** Sensitivity analysis for the association between CAR and all‐cause mortality with additional adjustment for eGFR.

Variables	Model 4	
	HR (95% CI)	*p* value
*CAR as a continuous variable*		
For every 1 unit increase	1.409 (1.216, 1.632)	< 0.001
For every 1 standard deviation increase	1.404 (1.213, 1.624)	< 0.001
*CAR as a categorical variable*		
Low CAR	Ref	
High CAR	1.418 (0.852, 2.361)	0.179

*Note:* Model 4: adjusted for age, BMI, SBP, UA, LDL‐C, Nt‐ProBNP, aspirin, clopidogrel, beta blocker, ARNI, and eGFR.

Abbreviations: ARNI, angiotensin receptor‐neprilysin inhibitor; BMI, body mass index; CAR, C‐reactive protein to albumin ratio; CI, confidence interval; eGFR, estimated Glomerular Filtration Rate; HR, hazard ratio; LDL‐C, low‐density lipoprotein cholesterol; NT‐proBNP, N‐terminal pro‐B‐type natriuretic peptide; Ref, reference; SBP, systolic blood pressure; UA, uric acid.

## 4 Discussion

In this single‐center retrospective cohort study of 342 patients with AECHF, we observed a significant independent association between the CAR and all‐cause mortality. After adjusting for potential confounders including age, BMI, SBP, uric acid, LDL‐C, NT‐proBNP, and multiple discharge medications, each unit increase in CAR was associated with a 41.2% higher risk of all‐cause mortality. Patients in the high CAR group (defined by the 85th percentile, CAR > 0.66) exhibited a 1.778‐fold increased risk compared with the low CAR group. The robustness of this association was further supported by subgroup analyses showing consistent findings across most strata, including age ≤ 75 years, age > 75 years, females, non‐smokers, patients with and without diabetes, with and without hypertension, without dyslipidemia, with atrial fibrillation, and without CHD. Furthermore, in the sensitivity analysis of further adjusting eGFR, for every 1 unit or 1 SD increase in CAR, the risk of all‐cause mortality still significantly increased. Kaplan–Meier survival analysis demonstrated a significantly lower cumulative survival rate in the high CAR group, indicating a worse prognosis, while ROC curve analysis confirmed a modest but statistically significant predictive value. These findings suggest that CAR, a readily available composite biomarker integrating inflammatory burden and nutritional status, may serve as a valuable tool for risk stratification in patients with AECHF.

The prognostic role of the CAR in cardiovascular diseases has gained increasing attention, yet evidence specifically focusing on the AECHF population remains limited. Systematic reviews have confirmed that CAR is significantly associated with both in‐hospital and out‐of‐hospital all‐cause mortality, higher rehospitalization rates, and advanced NYHA class in HF populations [17]. However, beyond its conventional interpretation as an inflammatory‐nutritional index, CAR—and particularly its denominator, albumin—integrates a far broader pathophysiological picture; it reflects systemic inflammation, hepatic congestion secondary to right‐sided HF, renal dysfunction with protein loss, capillary leakage, oxidative stress, and diminished physiological reserve [41]. In device‐treated HF patients, pre‐implantation hypoalbuminaemia independently predicts long‐term mortality and HF rehospitalisation following cardiac resynchronization therapy [42], and is similarly associated with incident atrial fibrillation and post‐implantation mortality in octogenarians with pacemakers [43]. The oxidative stress and systemic inflammatory activation underlying these chronic conditions are similarly pivotal in the pathophysiology of acute ischemic myocardial injury. In this regard, the uric acid/albumin ratio—a surrogate marker balancing oxidative stress against anti‐inflammatory reserve—has been demonstrated to independently predict microvascular dysfunction (no‐reflow phenomenon) in patients with ST‐elevation myocardial infarction [44]. Moreover, CAR itself has been validated as an independent predictor of long‐term mortality in HF with reduced ejection fraction patients with implantable cardioverter‐defibrillators [45], and exhibits superior discriminative ability for atrial fibrillation recurrence following cryoablation compared to other inflammatory indices [46]. These observations collectively underscore that CAR acts as a composite indicator of the intertwined processes of systemic inflammation and nutritional decline, rather than serving merely as a single diagnostic tool. This pathophysiological basis aligns closely with the biological mechanisms discussed below, which further elucidate how this inflammatory‐nutritional imbalance directly impacts myocardial homeostasis and patient prognosis. Building upon this pathophysiological framework, our study was specifically designed to validate the prognostic value of CAR in the AECHF population, where this inflammatory‐nutritional imbalance is most pronounced. Several aspects of our study warrant emphasis. First, by focusing on the acute exacerbation phase—a critical window characterized by heightened inflammatory activation and rapid nutritional deterioration—we directly addressed a notable evidence gap in this high‐risk setting. Second, our multivariate models incorporated a comprehensive range of confounders, including detailed discharge medications, thereby minimizing residual confounding. Third, comprehensive subgroup analyses demonstrated that the association between CAR and mortality remained stable across diverse clinical subgroups, particularly in females and non‐smokers, adding granularity to the existing literature.

Biologically, elevated Hs‐CRP directly reflects systemic inflammation driven by pro‐inflammatory cytokines (IL‐6, TNF‐α) and NLRP3 inflammasome activation, which promote myocardial remodeling and contractile dysfunction [47, 48]. Concurrently, hypoalbuminaemia impairs plasma colloid osmotic pressure, promotes capillary leakage, compromises clearance of inflammatory mediators, and exacerbates oxidative stress [41]. The combination of high Hs‐CRP and low albumin may synergistically activate apoptotic and autophagic pathways, heightening cellular vulnerability to stress [49]. Despite these plausible mechanisms, causal inference remains limited, and further mechanistic investigations are needed to delineate the underlying pathways.

Several limitations of this study should be acknowledged. First, despite the inclusion of a wide range of potential confounders in our multivariate models, we cannot exclude the possibility of residual or unmeasured confounding. Factors such as genetic predisposition, detailed dietary intake, socioeconomic status, occupational exposures, and frailty status were not available in this retrospective dataset and may influence both CAR levels and mortality risk. Additionally, we did not have systematic data on acute infectious burden or objective measures of hemodynamic congestion (e.g., clinical signs of fluid overload, inferior vena cava diameter). Since these factors can directly elevate Hs‐CRP and reduce albumin levels, their absence may lead to overestimation of the CAR‐mortality association. To address this, we performed a sensitivity analysis additionally adjusting for eGFR, which confirmed that the independent association for CAR remained robust when analyzed as a continuous variable. Notably, NYHA functional class was not adjusted in the sensitivity analysis. This decision was based on its lack of statistical significance in the univariate analysis and the inherently limited variability of NYHA class within our cohort, as the study primarily enrolled patients with NYHA class III–IV. Therefore, the potential influence of NYHA class could not be fully assessed, and future studies with broader NYHA distribution are needed. However, residual confounding from unmeasured variables cannot be entirely ruled out. However, residual confounding from unmeasured variables cannot be entirely ruled out. Second, this was a single‐center study with a relatively modest sample size, which may limit statistical power for certain subgroup analyses and reduce the generalizability of our findings to broader populations. The relatively wide CIs observed in some subgroup estimates reflect this limitation. To mitigate the risk of overfitting, we strictly adhered to the EPV principle, restricting Model 3 to 10 covariates given 95 mortality events. Nevertheless, a marginal EPV may still introduce minor overfitting, and we advocate for cautious interpretation. Third, It is equally important to critically appraise the prognostic role of CAR itself. Despite the statistically significant association observed in our study, the modest AUC of 0.578 clearly indicates that CAR alone has limited discriminative capacity for predicting all‐cause mortality in AECHF patients. This finding underscores that CAR should not be interpreted as a standalone predictive tool for independent clinical decision‐making. Its potential clinical utility lies in serving as an adjunctive and complementary component within a multiparametric risk assessment framework. When incorporated alongside established predictors such as NT‐proBNP, renal function, and echocardiographic parameters, CAR may provide incremental value for refining risk stratification and identifying patients with heightened inflammatory–nutritional imbalance who might otherwise be overlooked by conventional models. It is also noteworthy that we performed formal interaction tests for all subgroup analyses. Our results demonstrated that the association between CAR and all‐cause mortality was consistent across most subgroups, reinforcing the robustness of our findings. Notably, a significant interaction was observed for atrial fibrillation, where CAR exhibited a stronger prognostic effect in patients with atrial fibrillation. This finding suggests that the inflammatory‐nutritional imbalance reflected by CAR may play a particularly critical role in the pathophysiology of HF complicated by atrial fibrillation. However, given the exploratory nature of subgroup analyses and the relatively small sample size in certain strata, this interaction should be interpreted cautiously and warrants further validation in larger prospective studies. Fourth, as an observational study, we cannot establish causality between CAR and mortality; the observed association may reflect reverse causation or confounding by unmeasured disease severity. Fifth, CAR was measured only at admission, and we lacked serial measurements during follow‐up. Dynamic changes in CAR may provide incremental prognostic information beyond a single baseline value, as has been suggested for other inflammatory biomarkers in HF. Sixth, our primary outcome was all‐cause mortality, and we did not assess cause‐specific mortality (e.g., cardiovascular death vs. non‐cardiovascular death) or other clinically important endpoints such as rehospitalization, myocardial infarction, or stroke. Finally, although we used the 85th percentile to define high CAR based on our cohort distribution, there is no universally accepted cutoff value for CAR in AECHF, and the optimal threshold may vary by population characteristics. Future studies should aim to validate our findings in larger, multicenter, prospective cohorts with serial biomarker measurements and comprehensive outcome ascertainment.

## 5 Conclusion

In conclusion, this study demonstrates that higher CAR levels at admission are significantly and independently associated with an increased risk of all‐cause mortality in patients with AECHF. The association remained robust after extensive adjustment for confounders and across diverse subgroups. As CAR is derived from two routinely measured, inexpensive laboratory parameters (Hs‐CRP and albumin), it represents a practical and accessible tool for enhancing risk stratification in this vulnerable population. Given the increasing emphasis on precision medicine, incorporating CAR into clinical decision‐making may help identify high‐risk patients who could benefit from more intensive monitoring and tailored therapeutic interventions. However, further large‐scale, prospective, multicenter studies with serial biomarker assessments and standardized outcome definitions are warranted to validate our findings and to explore the potential impact of CAR‐guided management strategies on patient outcomes.

## Author Contributions

**Xi Gu:** conceptualization, methodology, software, investigation, data curation, formal analysis, visualization, writing − original draft. **Jinli Feng:** conceptualization, writing − review and editing. **Junfang Guo:** conceptualization, validation, funding acquisition, project administration, supervision, writing − review and editing. All authors read and approved the final manuscript.

## Ethics Statement

The study was approved by the Ethics Committee of Zhenjiang First People's Hospital. The research protocol conformed to the principles of the Declaration of Helsinki. All patients provided informed consent.

## Consent

The authors have nothing to report.

## Conflicts of Interest

The authors declare no conflicts of interest.

## Data Availability

The data used in this study can be obtained from the corresponding author upon reasonable request.
